# Disproportional enrichment of FoxP3^+^CD4^+^ regulatory T cells shapes a suppressive tumour microenvironment in head and neck squamous cell carcinoma

**DOI:** 10.1002/ctm2.753

**Published:** 2022-03-28

**Authors:** Seyeon Park, Chang Gon Kim, Dahee Kim, Min Hee Hong, Eun Chang Choi, Se‐Heon Kim, Young Min Park, Jinna Kim, Sun Ock Yoon, Gamin Kim, Sunhye Shin, Kyungsoo Kim, Yoon Woo Koh, Sang‐Jun Ha, Hye Ryun Kim

**Affiliations:** ^1^ Department of Biochemistry College of Life Science and Biotechnology Yonsei University Seoul Republic of Korea; ^2^ Division of Medical Oncology Department of Internal Medicine Yonsei Cancer Center Yonsei University College of Medicine Seoul Republic of Korea; ^3^ Department of Otorhinolaryngology Yonsei University College of Medicine Seoul Republic of Korea; ^4^ Department of Radiology Yonsei University College of Medicine Seoul Republic of Korea; ^5^ Department of Pathology Yonsei University College of Medicine Seoul Republic of Korea

Dear Editor,

The efficacy of PD‐1 inhibitors has fallen short in human papilloma virus (HPV)‐positive head and neck squamous cell carcinoma (HNSCC), underscoring the clinical relevance of exploring the mechanism shaping treatment resistance. In this study, the suppressive tumour microenvironment of HNSCC was investigated to gain insights into anti‐PD‐1 resistance and conceive the better treatment option.

Based on transcriptomic data derived from the Cancer Genome Atlas (TCGA),^1^ HNSCC had the highest frequency of the IFN‐γ–dominant subtype, with over 70% of cases classified as IFN‐γ‐dominant (Figure 1A). Since immune checkpoint blockade has shown modest efficacy in treating HNSCC,^2^, ^3^, ^4^, ^5^ we investigated the key mechanisms involved in shaping an immune‐suppressive tumour microenvironment that counterbalances the active IFN‐γ signature. We reasoned that the robust adaptive antitumour immune response is actively suppressed by a subpopulation of tumour‐infiltrating (TI) lymphocytes, and therefore investigated the expression of *FoxP3*, a specific marker for regulatory T cells (Tregs).^6^ Intriguingly, *FoxP3* expression was highest in the HNSCC cohort, along with the frequency of the IFN‐γ‐dominant subtype (Figures 1B and S1A), although the expression of *CD4*, *CD8A* and *CD8B* was intermediate in HNSCC (Figure S1B–D). Therefore, we focused on TI Tregs in HNSCC, which are hypothesised to underlie the key mechanisms involved in shaping an immunosuppressive microenvironment that results in checkpoint blockade resistance.

**FIGURE 1:**
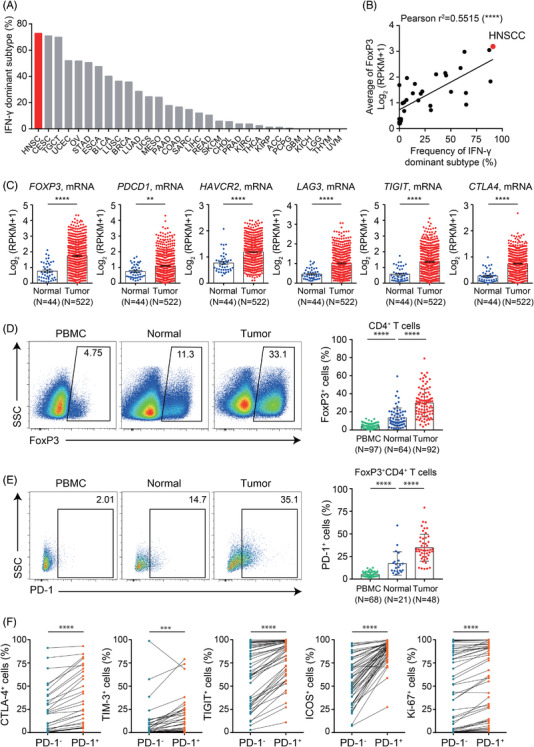
Disproportional enrichment of suppressive FoxP3^+^CD4^+^ regulatory T cells in the tumour microenvironment of HNSCC. (A) The proportions of IFN‐γ dominant subtypes were analysed from TCGA pan‐cancer transcriptome data. The IFN‐γ–dominant subtype was characterised by a strong CD8 signal, the greatest diversity of T‐cell receptors (TCRs), and the highest density of tumour‐infiltrating lymphocytes (TILs). This subtype also exhibited the highest expression levels of chemokines such as CXCL10 and CCL5. All of these traits confer a strong antitumour immune response, a prerequisite for immune checkpoint blockade response. Abbreviations for the cancer types were followed on the suggestions of the National Cancer Institute Genomic Data Commons (http://gdc.cancer.gov.). (B) Correlation between the frequency of IFN‐γ dominant subtypes and the expression of *FoxP3* mRNA from TCGA pan‐cancer cohort. RPKM, reads per kilobase per million. (C) Expression of *FoxP3, PDCD1, HAVCR2, LAG3, TIGIT* and *CTLA4* mRNA levels in normal tissue and tumour tissue were analysed from TCGA HNSCC cohort. (D)–(E) Flow cytometric analysis of TI CD4^+^ T cells or FoxP3^+^CD4^+^ regulatory T cells isolated from HNSCC patients who underwent surgical resection. Gating strategies for analysing the flow cytometry data are shown in Figure S2. (D) Representative flow cytometry plots display the expression of FoxP3 in CD4^+^ T cells from peripheral blood mononuclear cells (PBMCs), adjacent normal tissue and tumour tissue of HNSCC patients (left). The expression level of FoxP3^+^ cells gated on CD4^+^ T cells by tissue dependent sites (right). (E) Representative flow cytometry plots show the PD‐1^+^ populations of FoxP3^+^CD4^+^ Tregs from PBMCs, adjacent normal tissue, and tumour tissue of HNSCC patients (left). The frequency of PD‐1^+^ cells in FoxP3^+^CD4^+^ regulatory T cells from the aforementioned tissue sites of HNSCC patients (right). (F) Flow cytometric analysis of the expression of CTLA‐4, TIM‐3, TIGIT, ICOS and Ki67in TI FoxP3^+^CD4^+^ Tregs into those with PD‐1‐negative and positive populations. Percentage of expressing each molecule in the two subpopulations of TI Tregs. Each line in the graph indicates the same tumour tissue derived from each individual patient. SSC, side‐scattered light. **p < .01; ***p < .001; ****p < .0001. All statistical analyses were performed using paired and unpaired Student's *t* test

Transcriptomic analysis of HNSCC cohort from TCGA revealed that the expressions of *FoxP3* and immune checkpoints were higher in tumour tissue than in normal adjacent tissue (Figure 1C). Flow cytometry analysis of clinical samples of HNSCC patients confirmed that the frequency of Tregs was higher in tumour than in normal adjacent tissue or peripheral blood (Figures 1D and S2). Intriguingly, PD‐1^+^ cells were more frequently observed among Tregs in tumour tissue (Figure 1E). These PD‐1^+^ TI Tregs expressed immune checkpoint receptors and Ki‐67 more frequently than PD‐1^−^ TI Tregs (Figure 1F), suggesting that these Tregs impeded CD8^+^ T‐cell functionality (Figure S3).

Based on the previous findings of the prognostic implications of HPV status in HNSCC,^7^ we further classified patients in terms of HPV status and differential T‐cell infiltration. Consistent with the transcriptomic data (Figure S4), immunohistochemical analysis revealed that both CD8^+^ T cells and Tregs exhibited greater infiltration in HPV‐positive than in HPV‐negative HNSCC (Figures 2A and S5), with an apparent positive correlation between the infiltration rates of CD8^+^ T cells and Tregs (Figure 2B). HPV‐positive and CD8^high^ HNSCC patients demonstrated more favorable outcomes than HPV‐negative and CD8^low^ patients in terms of both DFS and OS in the overall population, whereas DFS and OS did not differ according to the abundance of TI FoxP3^+^ T cells (Figures 2C and S6A). Next, we conducted subgroup analyses according to HPV positivity. We observed no association between CD8^+^ T‐cell infiltration and outcome in the HPV‐negative group. Interestingly, FoxP3^high^ patients exhibited better survival outcomes in terms of DFS and OS than FoxP3^low^ patients in the HPV‐negative group; likewise, FoxP3/CD8^high^ patients had better prognoses than FoxP3/CD8^low^ patients in the HPV‐negative group (Figures 2D and S6B). In the HPV‐positive group, CD8^high^ patients displayed better survival outcomes than CD8^low^ patients in terms of both DFS and OS, whereas differences between outcomes based on the abundance of TI Tregs were not evident. The FoxP3/CD8^high^ group showed poor clinical outcomes, which was contrary to observations in HPV‐negative patients (Figures 2E and S6C). Collectively, these results indicated that the balance between TI CD8^+^ T cells and Tregs differentially impacted individual survival according to HPV status.

**FIGURE 2:**
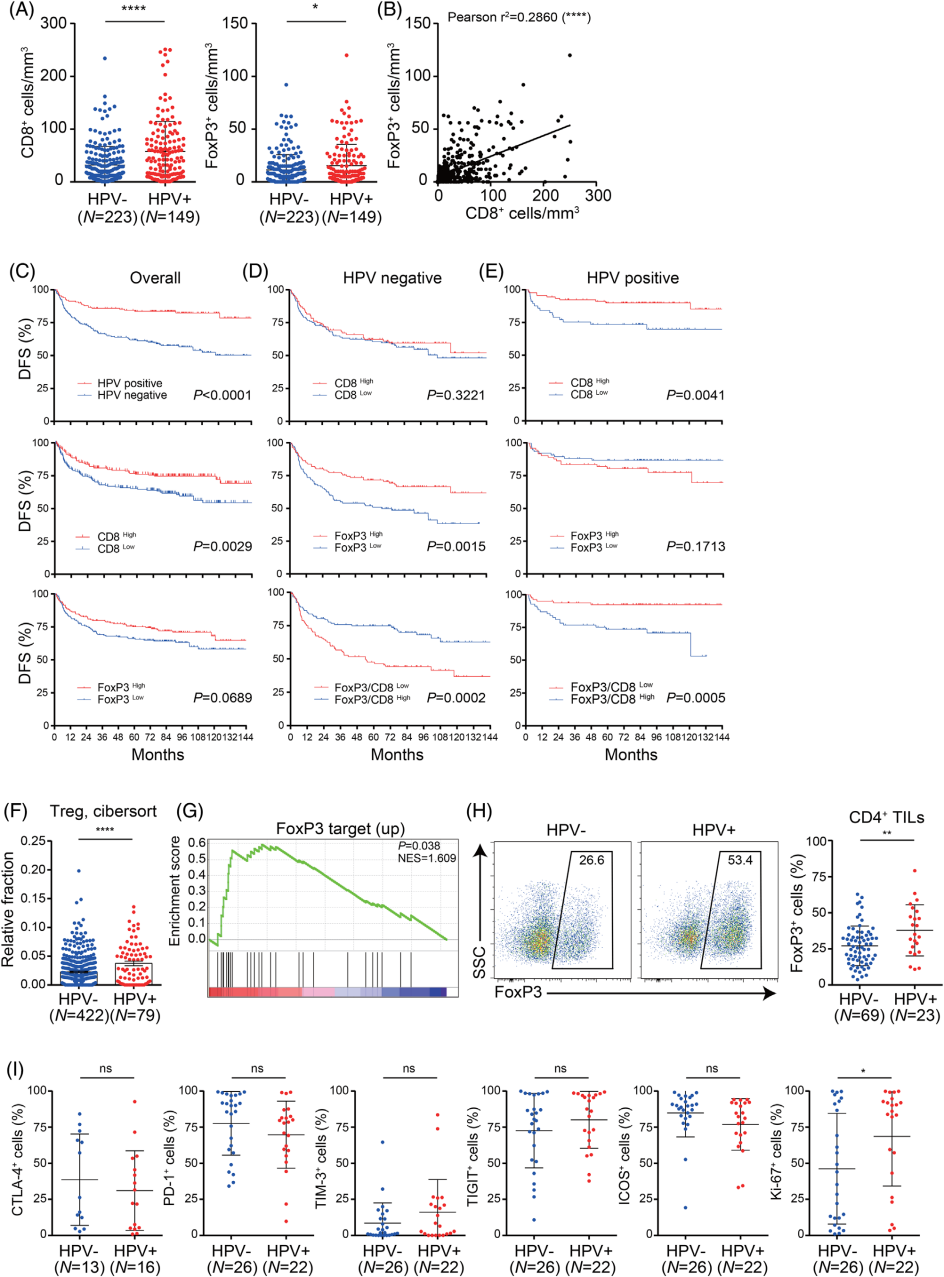
Disease‐free survival outcome and immune phenotypic characteristics according to HPV positivity. (A) Infiltration of CD8^+^ cells and FoxP3^+^ cells in HPV‐negative (*N *= 223) and HPV‐positive (*N *= 149) HNSCC and (B) the correlation between infiltration of CD8^+^ cells and FoxP3^+^ cells were measured by immunohistochemistry (IHC). Representative IHC images of CD8, FoxP3 and p16 in HNSCC tumour tissue were shown in Figure S5. (C) Disease‐free survival analysis according to HPV positivity, CD8^+^ cell infiltration and FoxP3^+^ cell infiltration in the overall patients. (D) Disease‐free survival analysis according to CD8^+^ cell infiltration, FoxP3^+^ cell infiltration and the ratio of FoxP3^+^ versus CD8^+^ cells in patients with HPV‐negative HNSCC. (E) Disease‐free survival analysis according to CD8^+^ cell infiltration, FoxP3^+^ cell infiltration and the ratio of FoxP3^+^ cell versus CD8^+^ cell in patients with HPV‐positive HNSCC. (F) Relative fraction of Tregs among total immune cells were analysed from TCGA HNSCC cohort by deconvolution method. (G) Gene set enrichment analysis (GSEA) of FoxP3 target gene sets in tumour tissues was performed using the transcriptomes from patients with HPV‐positive HNSCC versus those with HPV‐negative HNSCC. (H)–(I) The expression level of FoxP3 and immune checkpoint molecules was measured by flow cytometry in HPV‐positivity manners. (H) Representative flow cytometry plots are presented the fraction of FoxP3^+^ cells in TI CD4^+^ T cells following HPV positivity (left). Percentage of FoxP3‐expressing cells in the TI CD4^+^ T‐cell population according to HPV positivity (right). (I) The proportion of CTLA‐4^+^, PD‐1^+^, TIM‐3^+^, TIGIT^+^, ICOS^+^ and Ki‐67^+^ cells in the TI FoxP3^+^CD4^+^ regulatory T‐cell population according to HPV status. DFS, disease‐free survival. ns, not significant; *p < .05; **p < .01; ****p < .0001. Statistical analyses were performed (A) using unpaired Student's *t* test, (B) using Logistic regression, (C) through (E) using Long‐rank test and in (F) through (I) using unpaired Student's *t* test

To evaluate the differences in the properties between TI Tregs in HPV‐positive and HPV‐negative HNSCC, we first analysed Cibersort for TCGA HNSCC cohort, which revealed that the relative fraction of Tregs was larger in HPV‐positive than in HPV‐negative HNSCC (Figure 2F). Moreover, gene set enrichment analysis revealed that the expression of genes downstream of FoxP3 was significantly upregulated in HPV‐positive compared to that in HPV‐negative HNSCC (Figure 2G). To address whether Tregs shape the suppressive tumour microenvironment in a quantitative or qualitative manner, we assessed the frequency and characteristics of TI Tregs using per‐cell‐based analysis. Indeed, FoxP3^+^ Tregs were significantly enriched in HPV‐positive than in HPV‐negative HNSCC (Figure 2H), albeit the expression of immune checkpoint inhibitory receptors in TI Tregs did not differ significantly between two groups (Figure 2I). In contrast, the expression of Ki‐67 was slightly higher in TI Tregs from HPV‐positive than HPV‐negative tumours (Figure 2I). This trend was maintained when we analysed the frequency of immune checkpoint receptors co‐expressing in PD‐1^+^ cells among TI Tregs (Figure S7).

Next, we investigated TCGA HNSCC data to identify the expression of upstream regulators during Treg differentiation. The expression of *TGFB1* was higher in HPV‐negative HNSCC, whereas that of *TGFB2*, *TGFB3* and *IL10* did not differ based on HPV status (Figure 3A). In contrast, the expression of *IDO1* and *IDO2* was highly upregulated in HPV‐positive compared to that in HPV‐negative HNSCC (Figure 3A). Furthermore, the correlation between the expression of *IDO1* or *IDO2* and the relative frequency of Tregs among CD4^+^ T cells was stronger in HPV‐positive than in HPV‐negative HNSCC (Figure 3B), suggesting that IDO pathway activation can be one of the key mechanisms involved in Treg induction and maintenance of the HPV‐positive HNSCC tumour microenvironment. We further confirmed via single‐cell transcriptomic data analysis that IDO was highly expressed in dendritic cells from the tumour microenvironment (Figure S8A),^8^ as well as in CD14^+^HLA‐DR^+^CD11c^+^ mature dendritic cells from PBMCs upon stimulation (Figure S8B and C). Supporting the aforementioned findings, promising clinical activity was observed in a patient with HPV‐positive HNSCC in an ongoing study of combined treatment with the IDO inhibitor epacadostat and the PD‐1 inhibitor pembrolizumab (Figure 3C and D).

**FIGURE 3:**
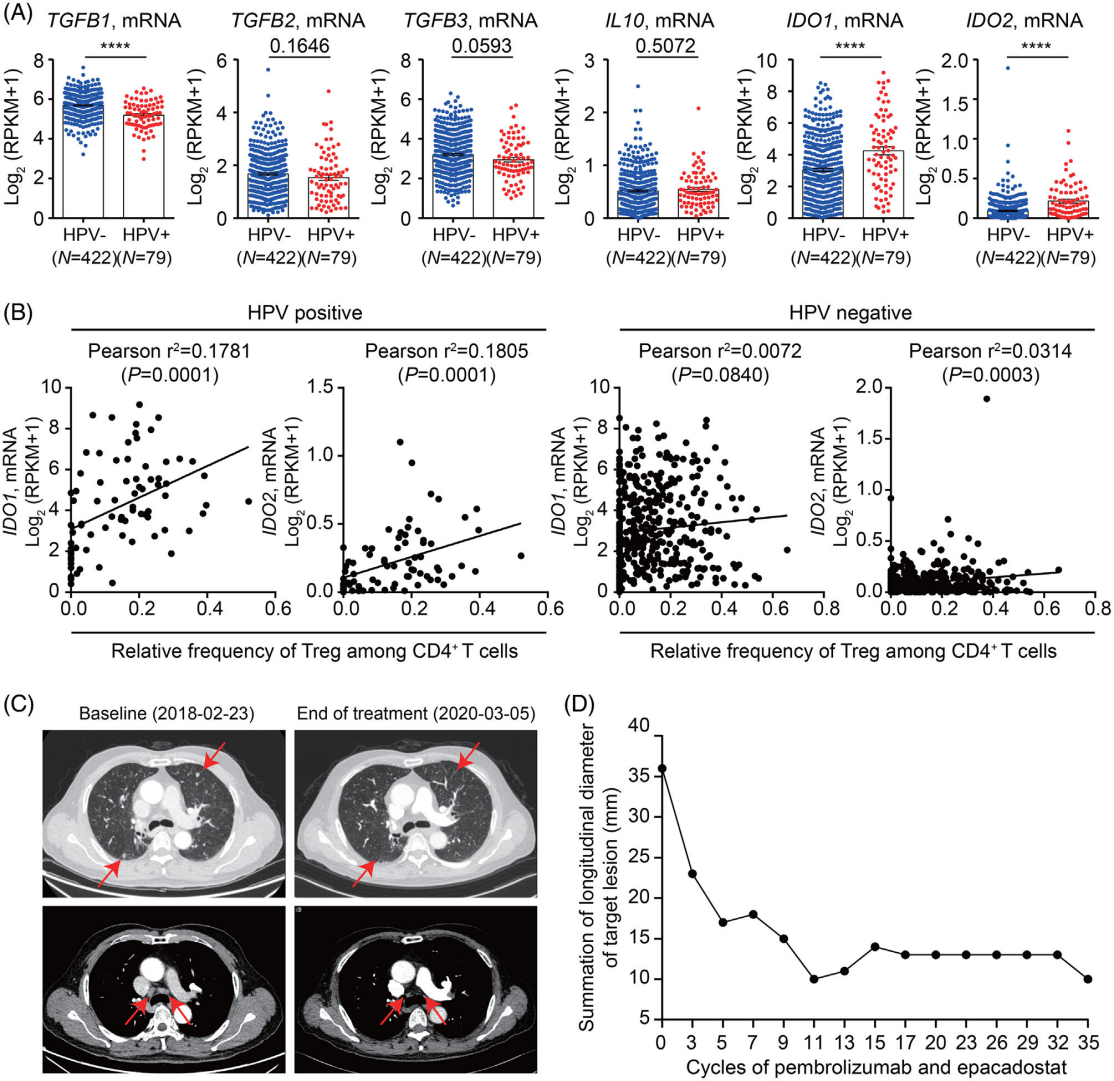
IDO pathway is involved in the enrichment of Tregs in HPV‐positive HNSCC and antitumour efficacy of combined anti‐PD‐1 and IDO inhibitor. (A) Expression of upstream regulators for Treg induction in HNSCC. Expression of *TGFB1*, *TGFB2*, *TGFB3*, *IL10*, *IDO1* and *IDO2* mRNA levels derived from TCGA HNSCC cohort according to HPV status. (B) Correlation between relative frequency of Treg among CD4^+^ T cells and the expression of *IDO1* and *IDO2* mRNA in the HPV‐positive and HPV‐negative patients from TCGA HNSCC cohort, respectively. (C), (D) Case study of antitumour efficacy of combined with pembrolizumab and epacadostat. A patient with oropharyngeal cancer with lung metastasis was enrolled in prospective trials (NCT03358472) and achieved partial response with more than 70% tumour reduction. This patient was progression‐free for 134 weeks, even after the scheduled discontinuation of epacadostat and pembrolizumab after completion of 35 cycles. (C) Representative CT scan images and (D) the summation of target lesion showing the reduction of tumour lesion. During the overall treatment course, the toxicity profiles of the combined IDO and PD‐1 inhibitors were manageable, without any dose interruption or discontinuation required. ****p < .0001. Statistical analyses were performed (A) using unpaired Student's *t* test and (B) using Pearson's correlation test

In conclusion, we identified that Tregs hamper active antitumour immunity in HNSCC, underlying anti‐PD‐1 resistance. In particular, activation of the IDO pathway contributes to the enrichment of Tregs in HPV‐positive HNSCC. A combined PD‐1 and IDO blockade elicited a consequential response in HPV‐positive HNSCC. Exploiting strategies to target Tregs or the IDO pathway would benefit patients with HPV‐positive HNSCC.

## CONFLICT OF INTEREST

The authors declare no potential conflicts of interest.

## Supporting information

SUPPORTING INFORMATION
