# Supplementary material for: Disproportional enrichment of FoxP3+CD4+ regulatory T cells shapes a suppressive tumour microenvironment in head and neck squamous cell carcinoma
Source: Clin Transl Med. 2022 Mar 28;12(3):e753. doi: 10.1002/ctm2.753 (PMC8958410; doi:10.1002/ctm2.753)
Supplement: Supplementary file 1 — SUPPORTING INFORMATION [file CTM2-12-e753-s001.docx]

**Supporting information for**

“Disproportional enrichment of regulatory T cells shapes a suppressive tumor microenvironment in head and neck squamous cell carcinoma”

Seyeon Park^1,#^, Chang Gon Kim^2,#^, Dahee Kim^3,#^, Min Hee Hong^2^, Eun Chang Choi^3^, Se-Heon Kim^3^, Young Min Park^3^, Jinna Kim^4^, Sun Ock Yoon^5^, Gamin Kim^2^, Sunhye Shin^2^, Kyungsoo Kim^1^, Yoon Woo Koh^3,*^, Sang-Jun Ha^1,*^ and Hye Ryun Kim^2,*^

**Text S1.** Method and material

**Text S2.** Discussion

**Text S3.** Reference for supporting information

**Table S1.** Baseline characteristics of patients

**Figure S1.** Expression of markers for T cell in TCGA pan cancer cohort according to cancer type

**Figure S2.** Representative gating strategies for analyzing flow cytometry data in PBMC and tumor tissue from HNSCC patients

**Figure S3.** Suppressive capacity of tumor-infiltrating Tregs from HPV-positive HNSCC

**Figure S4.** Expression of T cell transcriptome in TCGA HNSCC cohort according to HPV status

**Figure S5.** Representative immunohistochemical images of CD8, FoxP3 and p16 in HNSCC tumor tissue

**Figure S6.** Overall survival analysis according to HPV positivity, CD8^+^ T cell infiltration, FoxP3^+^ cell infiltration, and their balance

**Figure S7.**Phenotypic characteristics of tumor-infiltrating Tregs, FoxP3^-^CD4^+^ T cells and CD8^+^ T cells according to HPV status

**Figure S8.** Expression of IDO-1 and IDO-2 in immune cell subsets

**Figure S9.** Inhibition of IDO with epacadostat and Treg abundancy

**Figure S10.** Comparison of IDO1 and IDO2 expression and their correlation with Treg frequency between TCGA skin cutaneous melanoma (SKCM) and HPV-positive HNSCC cohort

**Figure S11.** Graphical abstract

**Text S1.** Method and material

**│ Sample preparation**

Fresh peripheral blood (N=97), normal adjacent tissue (N=64), and tumor tissue (N=92) samples were obtained from patients with HNSCC who underwent surgical resection between 2016 July and 2018 September (**Table S1**), who provided consent to enroll the following study. To isolate peripheral blood mononuclear cells (PBMCs), peripheral blood of HNSCC patients was mixed with RPMI containing 2% Fetal bovine serum (FBS; Biowest, Cat#S1480-500) and gently underlaid an equal volume of Histopaque-1077 (Merck, Cat#10771). PBMCs were isolated using density gradient centrifugation. After centrifugation, supernatant was removed using Pasteur pipette. Subsequently, the pelleted cells were treated with RBC lysis buffer to lysis any remaining red blood cells. To acquire lymphocytes from normal tissue and tumor tissue, tissues were minced into 1mm^3^ and digested with solution containing 1mg/ml collagenase type IV (Worthington Biochemical corp., Cat#LS004189) and 0.01mg/ml DNase I (Merck, Cat#D5025) at 37°C for 20 min. Dissociated tissues were filtered out using a 40μm cell strainer (Falcon, Cat#352340) into RPMI 1640 medium (Corning Inc., Cat#10-040-CVRC) and then centrifuged by Percoll gradient (Merck, Cat#P4937). Lymphocytes were obtained and suspended into PBS with 2% FBS. Ethical approval was obtained from Institutional Review Board (IRB) of Severance Hospital (4-2018-1210) and the study was conducted based on the tenets of the Declaration of Helsinki.

**│ Flow cytometry analysis”**

Single cell suspensions were analyzed using a FACS CytoFLEX (Beckman Coulter, Brea, CA) after staining with the following antibodies: Biolegend: CD3-BV785 (OKT3, Cat#317329), CD4-BV605 (RPA-T4, Cat#300556), CD8-BV605 (RPA-T8, Cat#301040), CD8-BV650 (RPA-T8, Cat#301042), CD11c-BV421 (S-HCL-3, Cat#371512), CD25-BV421 (BC96, Cat#302630), CD69-FITC (FN50, Cat#310904), PD-1-BV421 (EH12.2H7, Cat#329920), PD-L1 (29E.2A3, Cat#329714), TIM-3-PerCP-Cy5.5 (F38-2E2, Cat#345016), LAG-3-PE-Cy7 (11C3C65, Cat#369309), CTLA-4-PerCP-Cy5.5 (BNI3, Cat#369608), ICOS-BV510 (C398.4A, Cat#313525), Foxp3-PE (206D, Cat#320108), Ki-67-Alexa Fluor 700 (Ki-67, Cat#350530), PVR-PE (TX24, Cat#337508), IL17A-BV605 (BL168, Cat#512326), Mouse IgG2b, κ-BV421 (MPC-11, Cat#400342), Mouse IgG2a, κ-PE (MOPC-173, Cat#400212); R&D systems: TIM-3-Alexa Fluor 488 (344823, Cat#FAB2365G), TIGIT-APC (741182, Cat#FAB7898A); BD Biosciences: CD4-APC-H7 (L200, Cat#560837), CD25-PE-Cy7 (M-A251 , Cat#561405), HLA-DR-V500 (G46-6, Cat#561224) and Foxp3-Alexa Fluor 647 (259D/C7, Cat#560045). Invitrogen: IDO-APC (eyedio, Cat#17-9477-42). Dead cells were excluded by staining LIVE/DEAD™ Fixable Red Dead Cell Stain Kit (Invitrogen, Cat#L34971). For intracellular staining, the cells were fixed and permeabilized using Foxp3 fixation/permeabilization solution (eBioscience, Cat#00-5521-00) or BD Cytofix/Cytoperm fixation/permeabilization solution (BD Bioscience, Cat#554722) and then Foxp3, CLTA-4, IDO, IL17-A and Ki-67 were stained thereafter. To detect the expression of IL-17A, tumor tissue was stimulated with phorbol 12-myristate 13-acetate (PMA) and ionomycin in the presence of Golgi-Stop (BD Biosciences, Cat#554724) and Golgi-Plug (BD Bioscience, Cat#555029) for 6hours. The cells were analyzed using FlowJo software (Tree star). Gating strategies for analyzing the flow cytometry data are shown in **Figure S2**.

**│ Treg suppression assay**

HPV-positive HNSCC tissue sample preparation performed as described above. After dissociation of tumor tissue, we isolated regulatory T cell using CD4^+^CD25^+^CD127^dim/-^ Regulatory T Cell Isolation Kit (Miltenyi Biotec, Cat#130-094-775). Peripheral blood of HNSCC patients was obtained from matched patients undergoing surgery. Lymphocytes isolated from peripheral blood was described above and isolated CD8^+^ T cells using CD8^+^ T Cell Isolation Kit (Miltenyi Biotec, Cat#130-096-495). CD8^+^ T cells from peripheral blood were labelled with CellTrace™ Violet dye (Invitrogen, Cat#C34557) at 37 °C for 20 minutes and washed twice. Cell Trace Violet-labelled CD8^+^ T cells are referred to as responder T cells (Tresp). TI FoxP3^+^CD4^+^ Tregs from primary HNSCC tissue and responder CD8^+^ T cells were co-cultured with Dynabeads™ Human T-Activator CD3/CD28 (Gibco, Cat#11131D) in different ratios, as described. The cells were incubated at 37 °C and 5% CO2 for 4 days. Proliferation of CD8^+^ T cells were measured by flow cytometry (CytoFLEX, Beckman Coulter)

**│ Immunohistochemical (IHC) analysis**

To distinguish HPV positivity, p16 IHC was investigated according to the conventionally accepted criteria. Samples were determined as positive for p16 when strong and diffuse nuclear and cytoplasmic staining was observed in more than 70% of all HNSCC tumor cells, but was otherwise recorded as negative. The densities of TI CD8^+^ T cells and FoxP3^+^CD4^+^ Tregs were assessed by counting for the abundance of cells infiltrating tumor cell nests from five representative high-power fields under 400x magnifications. Preserved intact lymphocytes expressing CD8 or FoxP3 were counted manually, and the cell counts were averaged. Representative immunohistochemical images are shown in **Figure S5**.

**│ The Cancer Genome Atlas (TCGA) data analysis**

RNA-seq data from TCGA database were obtained from Firebrose (Broad Institute). The log2 (RPKM+1) values were obtained from each sample. The immune subtypes of each sample were categorized according to previous studies.^1^ Gene set enrichment analysis (GSEA) was used to test for enrichment of a specific gene set. A normalized enrichment score (NES) was specified for each GSEA.

**│ Detection of IDO expression derived from mature DC**

To detect the expression of IDO, we first generated monocyte-derived dendritic cells (MoDCs) from purified monocytes from PBMCs (N=5) using a CD14^+^ cell isolation kit (Miltenyi Biotec, Cat#130-050-201). We incubated CD14^+^ cells with the media containing human granulocyte-macrophage colony-stimulating factor (hGM-CSF) and rhIL-4 (Miltenyi Biotec, Cat#130-094-812) for 4 days to differentiate MoDCs. Differentiated MoDCs were stimulated at day 4 with either hIFN-g (10ng/ml) or LPS (100ng/ml and 2ug/ml) to differentiate into mature DC (mDC) for 24 hours. We measured the level of IDO from mDC by flow cytometry.

**│** **Generation of *in vitro*-derived induced Tregs (iTregs)**

To generate “induced” Tregs (iTregs), PBMCs derived from healthy donors were cultured in RPMI media (Corning Inc), supplemented with anti-CD3 (0.5ug/ml), anti-CD28 (0.5ug/ml), rhIL-2 (40ng/ml), and (with or without) rhTGF-β1 (5ng/ml) for 5 days. Differentiated cells were further incubated with either rhIL-2 (40ng/ml), Epacadostat (10 μg/mL, Sellekchem), or combination of rhIL-2/epacadostat for 14 days and changed media every 3 days. Cells were harvested and analyzed by flow cytometry.

**│ Induction of FoxP3^+^CD4^+^ T cells *in vitro***

PBMCs from HPV-positive HNSCC patients were incubated with rhIL-2 (200 IU/mL) either in the presence or absence of anti-human PD-1 antibody (10 μg/mL, EH12.2H7) or Epacadostat (10 μg/mL, Sellekchem). The media containing each agent was changed periodically every 3 days. After 14 days, cells were harvested and the expression of FoxP3 in CD4^+^ T cells were measured by flow cytometry. Fold change of FoxP3^+^ cells among CD4^+^ T lymphocytes relative to the control was calculated.

**│ Statistical analysis**

Statistical analysis was conducted with Prism 7 (GraphPad Software). Unless otherwise stated, a two-tailed paired or unpaired Student’s t-tests was analyzed to determine statistical significance (**P* < 0.05, ***P* < 0.01, ****P* < 0.001, *****P* < 0.0001). Data are expressed as means ± SEM. Pearson correlation analyses were used to evaluate correlations between variables. Survival was plotted using Kaplan-Meier curves and compared using the log-rank test.

**Text S2.** Discussion

In this study, we investigated how HPV infection affects the HNSCC tumor microenvironment and contributes to PD-1 blockade resistance. Although the IFN-γ–dominant subtype comprises the majority of HNSCC cases, the disproportional enrichment of FoxP3^+^CD4^+^ Tregs antagonized the antitumor immunity and was associated with a lower probability of responding to PD-1 blockade treatment. This feature was more evident in HPV-positive HNSCC cases, which were represented by an abundance of FoxP3^+^CD4^+^ Tregs associated with prognostic implications. In addition, the IDO pathway was involved in Treg induction in HPV-positive HNSCC. Promising antitumor efficacy was observed when PD-1 and IDO-1 inhibitors were combined. These findings collectively shed new light on immunologic characteristics of HNSCC with pathophysiologic and therapeutic relevance.

An estimated 10–15% of cancers are presumed to be related to viral infection.^2^ Currently, at least eight viruses, including Epstein-Barr virus (EBV), Merkel cell polyomavirus (MCV), and HPV have been associated with human cancers.^3^ Particularly, EBV, MCV, and HPV infections directly cause cancers such as nasopharyngeal cancer, gastric cancer, Merkel cell carcinoma, cervical cancer, anal cancer, and HNSCC. Virus-induced cancers exhibit a distinct tumor microenvironment with immunogenic potential.^4^ For instance, the PD-1/PD-L1 blockade has shown promising efficacy in EBV-associated gastric cancer,^5,6^ nasopharyngeal cancer,^7^ and Merkel cell carcinoma.^8,9^ In contrast, HPV-positive HNSCC exhibited a lower response rate than expected,^10-12^ despite exhibiting distinct characteristics compared with its HPV-negative counterpart. Correspondingly, using HPV status to dictate the use of PD-1 blockade immunotherapy for HNSCC is not recommended.^13^

To explore the fundamental mechanisms involved in this disparity, we investigated cell populations with immunosuppressive properties enriched in the HNSCC tumor microenvironment and found that Tregs played an essential role in promoting anti–PD-1 resistance in HPV-positive HNSCC. By leveraging TCGA dataset, we found that HNSCC, especially HPV-positive HNSCC, had the highest level of FoxP3 expression. Intrinsic resistance to PD-1 blockade was attributed to this trait, despite a high density of tumor-infiltrating immune cells. This result was confirmed by flow cytometry analysis of TILs; PD-1^+^ populations constituted the main proportion of TI Tregs, whereas Tregs from normal adjacent mucosa or peripheral blood featured lower proportions of PD-1^+^ populations. The suppressive functionality of TI Tregs was demonstrated via co-culture assay of TI FoxP3^+^CD4^+^ Tregs and autologous CD8^+^ T cells (**Figure S3A** and **S3B**). Moreover, we previously reported the suppressive function of PD-1^+^ and PD-1^-^ Treg, which showed that PD-1^high^ TIL Treg significantly suppressed CD8^+^ T cells compared to PD-1^low^ PBL Treg or Spleen Treg in NSCLC patient and TC-1 injected mouse, respectively.^14^ Given that the reinforcement of tumor-specific CD8^+^ T cell reactivity is the crucial component of PD-1 blockade, our experiment demonstrated that TI Tregs in HNSCC reduced the antitumor immunity mediated by effector T cells, inducing anti–PD-1 resistance. Moreover, the balance between TI Tregs and CD8^+^ T cells was associated with the prognosis of patients with HPV-positive HNSCC after surgical resection. Thus, we reasoned that targeting TI Tregs can be a promising strategy to facilitate the antitumor immunity mediated by tumor-specific effector T cells.

Several factors are known to be involved in Treg differentiation and function, including the TGF-β, IL-2, IL-10, and IDO families.^15-17^ Among these regulators, IDO-1 and -2 were uniquely upregulated in HPV-positive HNSCC, which can be induced by inflammatory cues accompanying HPV infection.^18,19^ Intriguingly, IDO-1 inhibitor epacadostat reduced fate decision toward Treg during *in vitro* differentiation (**Figure S9**). Of note, robust antitumor efficacy was observed in a patient with HNSCC treated with combined PD-1 and IDO inhibitors. This result was supported by the finding that reprogrammed Tregs, under conditions of IDO ablation, convert uniformly into a phenotype resembling proinflammatory Th17 cells, expressing IL-17, IL-2, and tumor necrosis factor-α.^20,21^ Collectively, we identified that IDO pathway–mediated Treg accumulation shaped the suppressive tumor microenvironment of HPV-positive HNSCC, with clinical implications.

Increased tryptophan metabolism by the IDO pathway induces immunosuppression, suggesting that cancer cells utilize this pathway to evade immune surveillance.^17^ In particular, IDO-1 and -2 have various effects on immune cells, including inactivation of effector T cells and NK cells, stimulation of Tregs, promotion of tolerogenic dendritic cells, and expansion of myeloid-derived suppressor cells.^22-24^ Based on these traits, IDO inhibitors including epacadostat, navoximod, and indoximod have been actively developed.^25^ However, the ECHO-301/KEYNOTE-252 study (NCT02752074) testing the combination of epacadostat and pembrolizumab as treatment for patients with advanced melanoma failed to meet its primary endpoints.^26^

When we analyzed the reasons for this failure, we were able to propose several biological hypotheses. First, the ECHO-301/KEYNOTE-252 study was conducted without biomarker enrichment in patients with melanoma, in which IDO-1 and -2 expression is intermediate. Furthermore, expression of the IDO family is heterogeneous at the intra- and inter-patient levels.^27^ For example, we found that IDO-1 and -2 expression in melanoma tissue was lower than that in HPV-positive HNSCC tissue (**Figure S10A**), providing evidence that IDO pathway inhibition is not as compelling in melanoma as it is in HPV-positive HNSCC. Second, IDO-1 and -2 expression was not as strictly correlated with the relative frequency of Tregs in melanoma as it was in HPV-positive HNSCC (**Figure S10B**), suggesting that the contribution of the IDO pathway to Treg induction in the tumor microenvironment was relatively smaller in patients with melanoma. Based on this hypothesis, we propose that targeting Tregs via IDO pathway inhibition can be a promising strategy to augment antitumor immunity in HPV-positive HNSCC. A subgroup analysis of the ECHO-304/KEYNOTE-669 study that tests the combination of epacadostat and pembrolizumab as treatment for patients with R/M HNSCC according to HPV status is of interest for the future.

Our study has some limitations. First, our analysis was mainly conducted with samples derived from patients not treated with PD-1 blockade, limiting clinical significance. Second, suppressive roles of Tregs and prognostic value of HPV were relatively well known, although we investigated mechanistic link between IDO pathway activation and induction of Tregs and proved preclinical relevance of targeting IDO pathway. Third, pathways or populations involved in hampering antitumor immunity were not comprehensively explored and there would be possibilities that IDO pathway activation and Treg enrichment are not the major factors involved in immune suppression in HNSCC. Currently ongoing studies exploiting IDO inhibitors (NCT03358472 and NCT03854032) would clearly address whether targeting of IDO pathway activation and Treg are relevant approach in HNSCC.

In summary, we demonstrated that HNSCC possesses a distinct immune microenvironment with a disproportional enrichment of TI FoxP3^+^CD4^+^ Tregs, especially in HPV-positive HNSCC. TI FoxP3^+^CD4^+^ Tregs diminished the antitumor immunity mediated by CD8^+^ T cells, with prognostic implications. Furthermore, the IDO pathway was identified as a key player in Treg-mediated anti–PD-1 resistance, and targeting the IDO pathway circumvented anti–PD-1 resistance in HPV-positive HNSCC. Based on our results, we propose that the pharmacologic inhibition of the IDO pathway diminishes Treg abundance and that combining IDO-1 inhibition with anti–PD-1 therapy may be beneficial for patients with HPV-positive HNSCC (**Figure S11**). Ongoing clinical studies of this strategy may support our findings. In addition, the effect of IDO pathway inhibition on the regulation of tumor-specific CD8^+^ T lymphocytes requires further clinical investigation with clinical samples from patients treated with IDO inhibitors.

**Text S3.** Reference for supporting information

1. Thorsson V, Gibbs DL, Brown SD, et al. The Immune Landscape of Cancer. *Immunity.* 2018;48(4):812-830.e814.

2. Mesri EA, Feitelson MA, Munger K. Human viral oncogenesis: a cancer hallmarks analysis. *Cell Host Microbe.* 2014;15(3):266-282.

3. de Martel C, Georges D, Bray F, Ferlay J, Clifford GM. Global burden of cancer attributable to infections in 2018: a worldwide incidence analysis. *Lancet Glob Health.* 2020;8(2):e180-e190.

4. Varn FS, Schaafsma E, Wang Y, Cheng C. Genomic Characterization of Six Virus-Associated Cancers Identifies Changes in the Tumor Immune Microenvironment and Altered Genetic Programs. *Cancer Res.* 2018;78(22):6413-6423.

5. Kim ST, Cristescu R, Bass AJ, et al. Comprehensive molecular characterization of clinical responses to PD-1 inhibition in metastatic gastric cancer. *Nat Med.* 2018;24(9):1449-1458.

6. Panda A, Mehnert JM, Hirshfield KM, et al. Immune Activation and Benefit From Avelumab in EBV-Positive Gastric Cancer. *J Natl Cancer Inst.* 2018;110(3):316-320.

7. Ma BBY, Lim WT, Goh BC, et al. Antitumor Activity of Nivolumab in Recurrent and Metastatic Nasopharyngeal Carcinoma: An International, Multicenter Study of the Mayo Clinic Phase 2 Consortium (NCI-9742). *J Clin Oncol.* 2018;36(14):1412-1418.

8. Nghiem PT, Bhatia S, Lipson EJ, et al. PD-1 Blockade with Pembrolizumab in Advanced Merkel-Cell Carcinoma. *N Engl J Med.* 2016;374(26):2542-2552.

9. Kaufman HL, Russell J, Hamid O, et al. Avelumab in patients with chemotherapy-refractory metastatic Merkel cell carcinoma: a multicentre, single-group, open-label, phase 2 trial. *Lancet Oncol.* 2016;17(10):1374-1385.

10. Seiwert TY, Burtness B, Mehra R, et al. Safety and clinical activity of pembrolizumab for treatment of recurrent or metastatic squamous cell carcinoma of the head and neck (KEYNOTE-012): an open-label, multicentre, phase 1b trial. *Lancet Oncol.* 2016;17(7):956-965.

11. Bauml J, Seiwert TY, Pfister DG, et al. Pembrolizumab for Platinum- and Cetuximab-Refractory Head and Neck Cancer: Results From a Single-Arm, Phase II Study. *J Clin Oncol.* 2017;35(14):1542-1549.

12. Cohen EEW, Soulières D, Le Tourneau C, et al. Pembrolizumab versus methotrexate, docetaxel, or cetuximab for recurrent or metastatic head-and-neck squamous cell carcinoma (KEYNOTE-040): a randomised, open-label, phase 3 study. *Lancet.* 2019;393(10167):156-167.

13. Cohen EEW, Bell RB, Bifulco CB, et al. The Society for Immunotherapy of Cancer consensus statement on immunotherapy for the treatment of squamous cell carcinoma of the head and neck (HNSCC). *J Immunother Cancer.* 2019;7(1):184.

14. Kim HR, Park HJ, Son J, et al. Tumor microenvironment dictates regulatory T cell phenotype: Upregulated immune checkpoints reinforce suppressive function. *J Immunother Cancer.* 2019;7(1):339.

15. Li MO, Rudensky AY. T cell receptor signalling in the control of regulatory T cell differentiation and function. *Nat Rev Immunol.* 2016;16(4):220-233.

16. Josefowicz SZ, Lu LF, Rudensky AY. Regulatory T cells: mechanisms of differentiation and function. *Annu Rev Immunol.* 2012;30:531-564.

17. Munn DH, Mellor AL. IDO in the Tumor Microenvironment: Inflammation, Counter-Regulation, and Tolerance. *Trends Immunol.* 2016;37(3):193-207.

18. Mittal D, Kassianos AJ, Tran LS, et al. Indoleamine 2,3-dioxygenase activity contributes to local immune suppression in the skin expressing human papillomavirus oncoprotein e7. *J Invest Dermatol.* 2013;133(12):2686-2694.

19. Krishna S, Ulrich P, Wilson E, et al. Human Papilloma Virus Specific Immunogenicity and Dysfunction of CD8(+) T Cells in Head and Neck Cancer. *Cancer Res.* 2018;78(21):6159-6170.

20. Sharma MD, Hou DY, Liu Y, et al. Indoleamine 2,3-dioxygenase controls conversion of Foxp3+ Tregs to TH17-like cells in tumor-draining lymph nodes. *Blood.* 2009;113(24):6102-6111.

21. Baban B, Chandler PR, Sharma MD, et al. IDO activates regulatory T cells and blocks their conversion into Th17-like T cells. *J Immunol.* 2009;183(4):2475-2483.

22. Labadie BW, Bao R, Luke JJ. Reimagining IDO Pathway Inhibition in Cancer Immunotherapy via Downstream Focus on the Tryptophan-Kynurenine-Aryl Hydrocarbon Axis. *Clin Cancer Res.* 2019;25(5):1462-1471.

23. Liu M, Wang X, Wang L, et al. Targeting the IDO1 pathway in cancer: from bench to bedside. *J Hematol Oncol.* 2018;11(1):100.

24. Cheong JE, Sun L. Targeting the IDO1/TDO2-KYN-AhR Pathway for Cancer Immunotherapy - Challenges and Opportunities. *Trends Pharmacol Sci.* 2018;39(3):307-325.

25. Prendergast GC, Malachowski WP, DuHadaway JB, Muller AJ. Discovery of IDO1 Inhibitors: From Bench to Bedside. *Cancer Res.* 2017;77(24):6795-6811.

26. Long GV, Dummer R, Hamid O, et al. Epacadostat plus pembrolizumab versus placebo plus pembrolizumab in patients with unresectable or metastatic melanoma (ECHO-301/KEYNOTE-252): a phase 3, randomised, double-blind study. *Lancet Oncol.* 2019;20(8):1083-1097.

27. Gide TN, Allanson BM, Menzies AM, et al. Inter- and intrapatient heterogeneity of indoleamine 2,3-dioxygenase expression in primary and metastatic melanoma cells and the tumour microenvironment. *Histopathology.* 2019;74(6):817-828.

**Table S1.** Baseline characteristics of patients

| **Variables** | | **Total patients (*N*=97)** | **Percentage** |
| --- | --- | --- | --- |
| Age | |  |  |
|  | Median (range) | 62 (31-85) | |
| Sex | |  |  |
|  | Male | 74 | 76.3 |
|  | Female | 23 | 23.7 |
|  |  |  |  |
| Smoking status | |  |  |
|  | Current | 40 | 41.2 |
|  | Former | 18 | 18.6 |
|  | Never | 39 | 40.2 |
|  |  |  |  |
| Human papilloma virus infection | |  |  |
|  | Positive | 41 | 42.3 |
|  | Negative | 56 | 57.7 |
|  |  |  |  |
| Stage | |  |  |
|  | I | 7 | 7.2 |
|  | II | 19 | 19.6 |
|  | III | 19 | 19.6 |
|  | IV | 52 | 53.6 |
|  |  |  |  |
| Lymphovascular invasion | |  |  |
|  | Positive | 28 | 28.9 |
|  | Negative | 69 | 71.1 |
|  |  |  |  |
| Perineural invasion | |  |  |
|  | Positive | 33 | 34.0  6 |
|  | Negative | 64 | 66.0 |
|  |  |  |  |
| Resection margin | |  |  |
|  | Positive | 42 | 43.3 |
|  | Negative | 55 | 56.7 |
|  |  |  |  |
| Extranodal extnesion | |  |  |
|  | Positive | 30 | 30.9 |
|  | Negative | 67 | 69.1 |


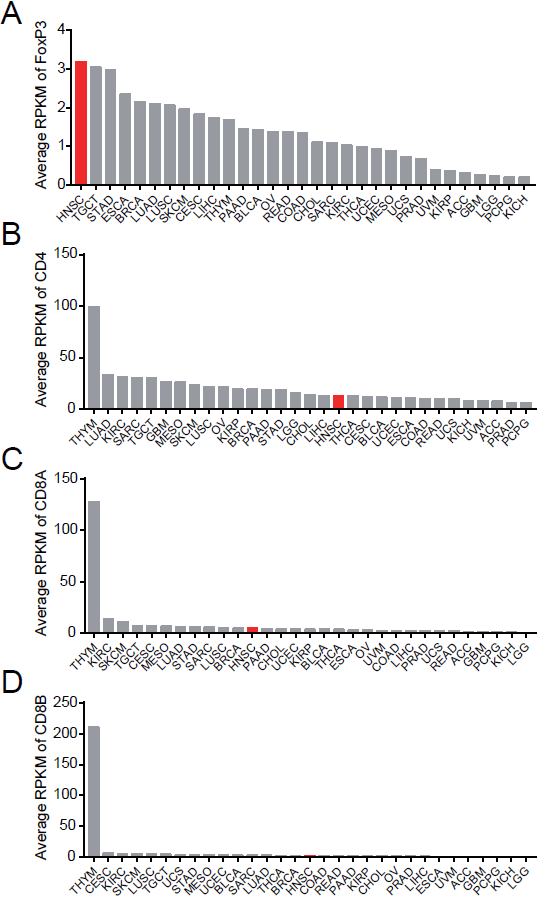


**Figure S1. Expression of T cell markers in TCGA pan cancer cohort according to cancer type.** Expression of **(A)** *FoxP3*, **(B)** *CD4*, **(C)** *CD8A* and **(D)** *CD8B* mRNA according to TCGA pan-cancer cohort. RPKM, reads per kilobase per million. ACC. Adrenocortical carcinoma; BLCA, Bladder Urothelial Carcinoma; BRCA, Breast invasive carcinoma; CESC, Cervical squamous cell carcinoma and endocervical adenocarcinoma; CHOL, Cholangiocarcinoma; COAD, Colon adenocarcinoma; ESCA, Esophageal carcinoma; GBM, Glioblastoma multilforme; HNSC, Head and Neck squamous cell carcinoma; KICH, Kidney Chromophobe; KIRC, Kidney renal clear cell carcinoma; KIRP, Kidney renal papillary cell carcinoma; LGG, Brain Lower Grade Glioma; LIHC, Liver hepatocellular carcinoma; LUAD, Lung adenocarcinoma; LUSC, Lung squamous cell carcinoma; MESO, Mesothelioma; OV, Ovarian serous cystadenocarcinoma; PAAD, Pancreatic adenocarcinoma; PCPG, Pheochromocytoma and Paraganglioma; PRAD, Prostate adenocarcinoma; READ, Rectum adenocarcinoma; SARC, Sarcoma; SKCM, Skin Cutaneous Melanoma; STAD, Stomach adenocarcinoma; TGCT, Testicular Germ Cell Tumors; THCA, Thyroid carcinoma; THYM, Thymoma; UCEC, Uterine Corpus Endometrial Carcinoma; UCS, Uterine Carcinosarcoma.


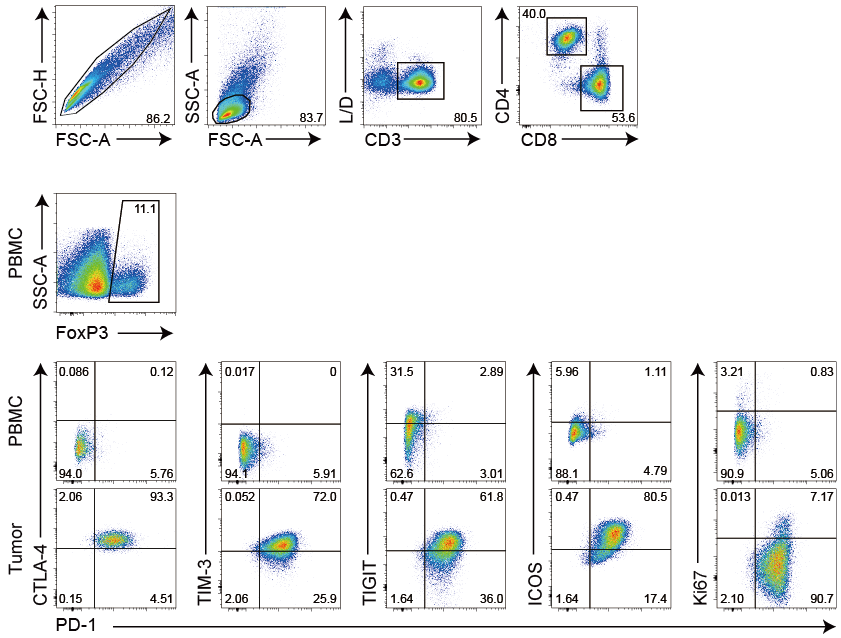


**Figure S2. Representative gating strategies for analyzing flow cytometry data in PBMC and tumor tissue from HNSCC patients.** Single cells firstly gated by forward scatter-area (FSC-A) against forward scatter-height (FSC-H). Lymphocytes were identified by forward scatter-area (FSC-A) versus side scatter-area (SSC-A) plot. And then the live CD3^+^ T cells were gating with CD3 antibody and LIVE/DEAD™ Stain Kit. The CD3^+^ T cells were subgated into CD4^+^ and CD8^+^ T cells. Representative expression of FoxP3 of CD4^+^ T cells, immune checkpoint molecules or activation markers such as PD-1, CTLA-4, TIM-3, TIGIT, ICOS and Ki67 in Tregs of PBMC and tumor tissue from HNSCC patients.


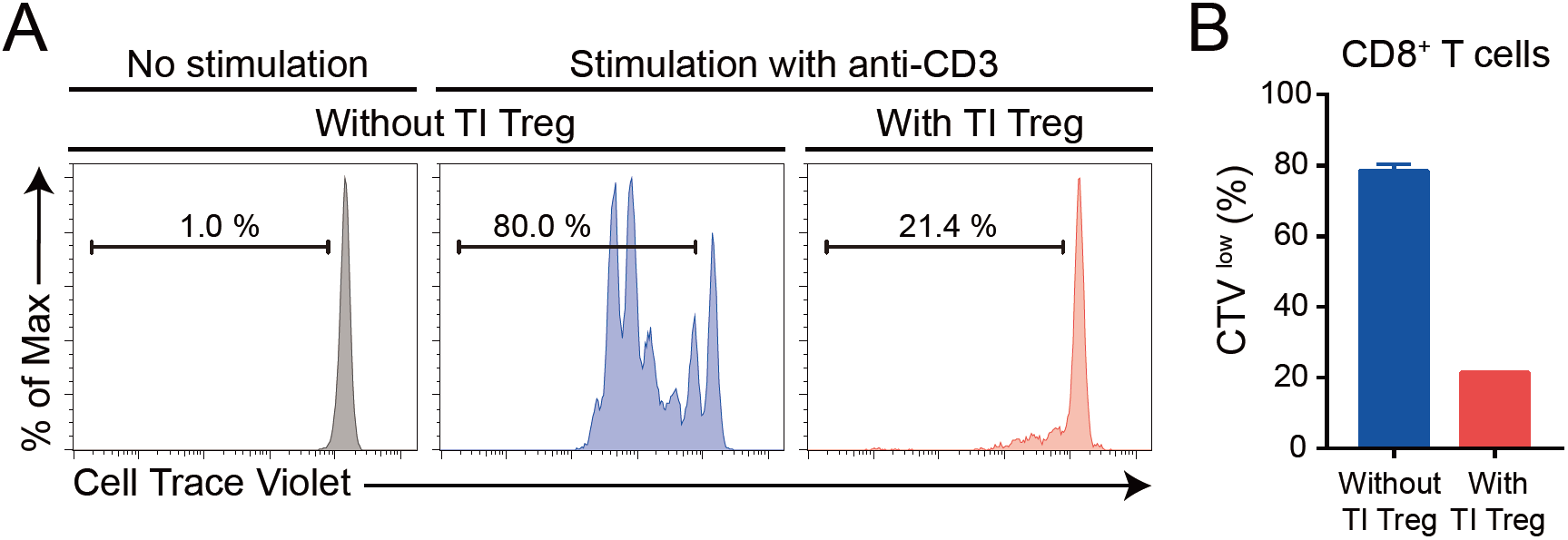


**Figure S3. Suppressive capacity of tumor-infiltrating T regs from HPV-positive HNSCC. (A)** Histogram representing the Cell Trace Violet (CTV) profile showing the proliferation gated on CD8^+^ T cells in the absence or presence of TI FoxP3^+^CD4^+^ Tregs derived from HPV-positive HNSCC. **(B)** Proliferation was analyzed by gating for CTV-labeled CD8^+^ T cells. Data shown in (A) and (B) are representative of four independent experiments.


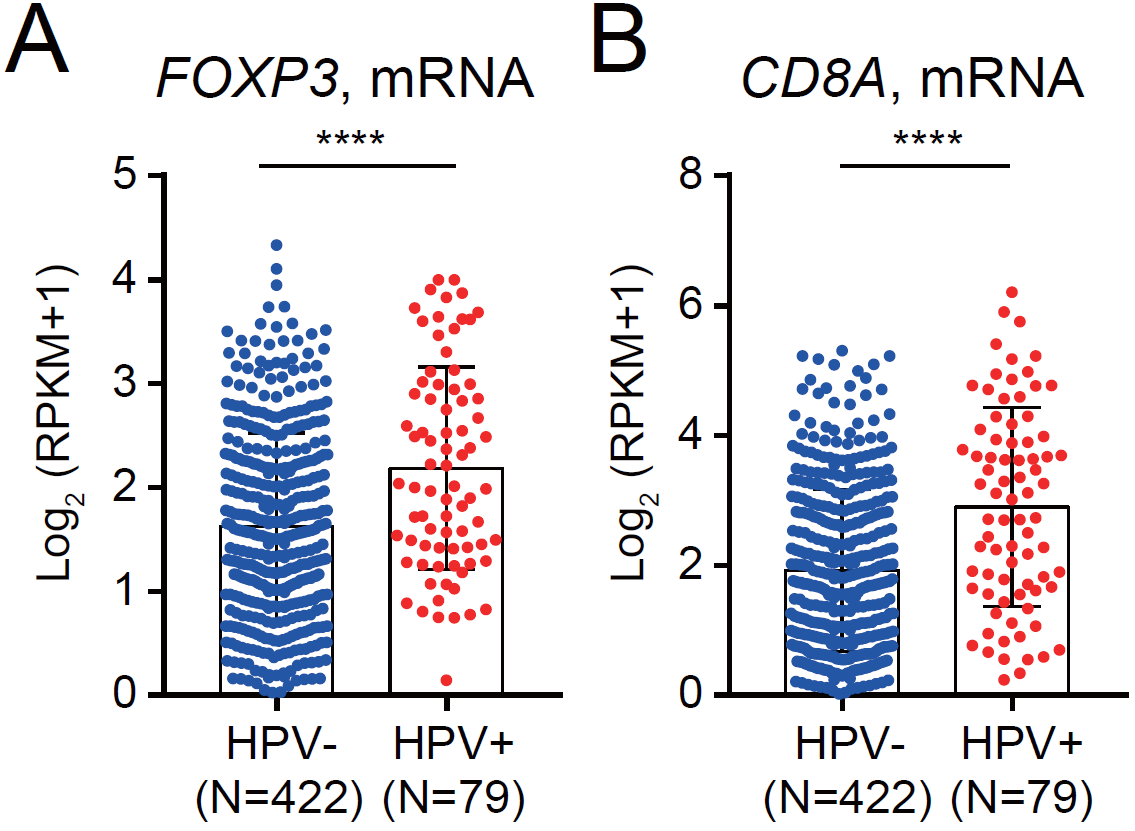


**Figure S4. Expression of the T cell transcriptome in TCGA HNSCC cohort according to HPV status. (A-B)** Expression of **(A)** *FoxP3* and **(B)** *CD8A* mRNA level in tumor tissues from TCGA HNSCC cohort according to HPV status. *****P* < 0.0001. All statistical analyses were performed using unpaired Student’s t test.


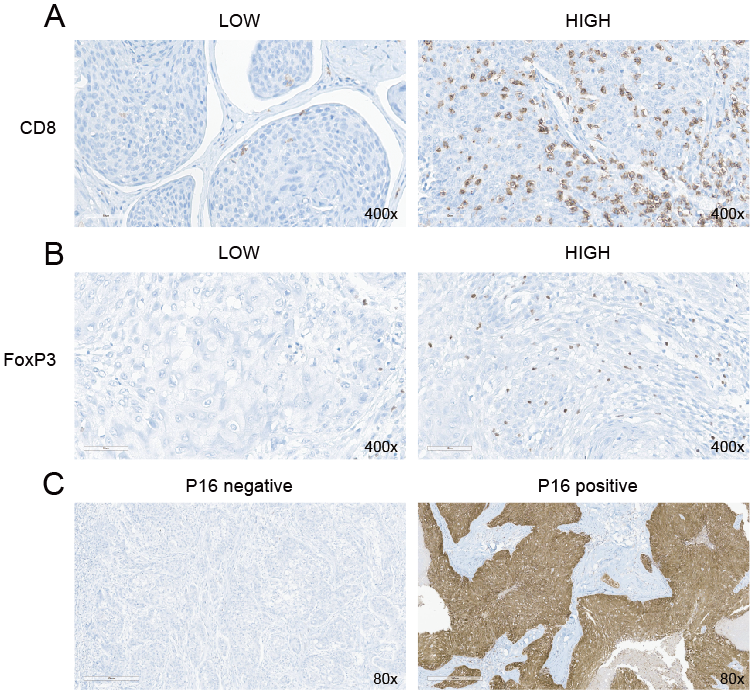
 **Figure S5. Representative immunohistochemical images of CD8, FoxP3 and p16 in HNSCC tumor tissue.** Immunohistochemical expression levels of **(A)** CD8 and **(B)** FoxP3 in HNSCC tumor tissue. **(C)** HNSCC tumor tissue stained for p16 to identify HPV positivity. Images were taken under x80 and x400 magnification for each field.


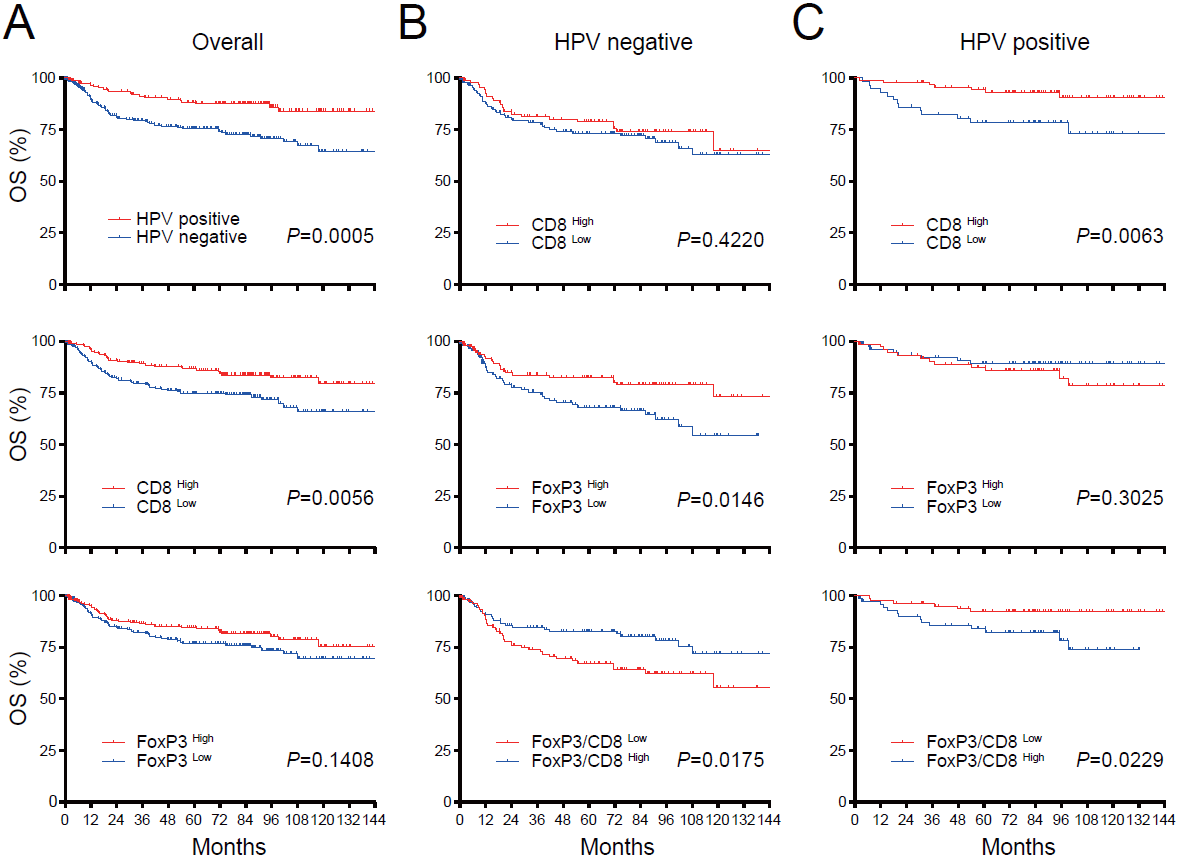


**Figure S6. Overall survival analysis according to HPV positivity, CD8^+^ T cell infiltration, FoxP3^+^ cell infiltration, and their balance. (A)** Overall survival analysis showing the outcome of all patients according to HPV positivity, CD8^+^ cell infiltration, and FoxP3^+^ cell infiltration in the HNSCC patients. **(B)** Overall survival curves according to CD8^+^ cell infiltration, FoxP3^+^ cell infiltration, and the ratio of FoxP3^+^ cell versus CD8^+^ T cell in patients with HPV-negative HNSCC. **(C)** Kaplan-Meier survival plots showing the overall survival analysis with the CD8^+^ cell infiltration, FoxP3^+^ cell infiltration and the ratio between FoxP3^+^ cell versus CD8^+^ cell in the patients with HPV-positive HNSCC. All statistical analyses were performed in (A) through (C) using Long-rank test.


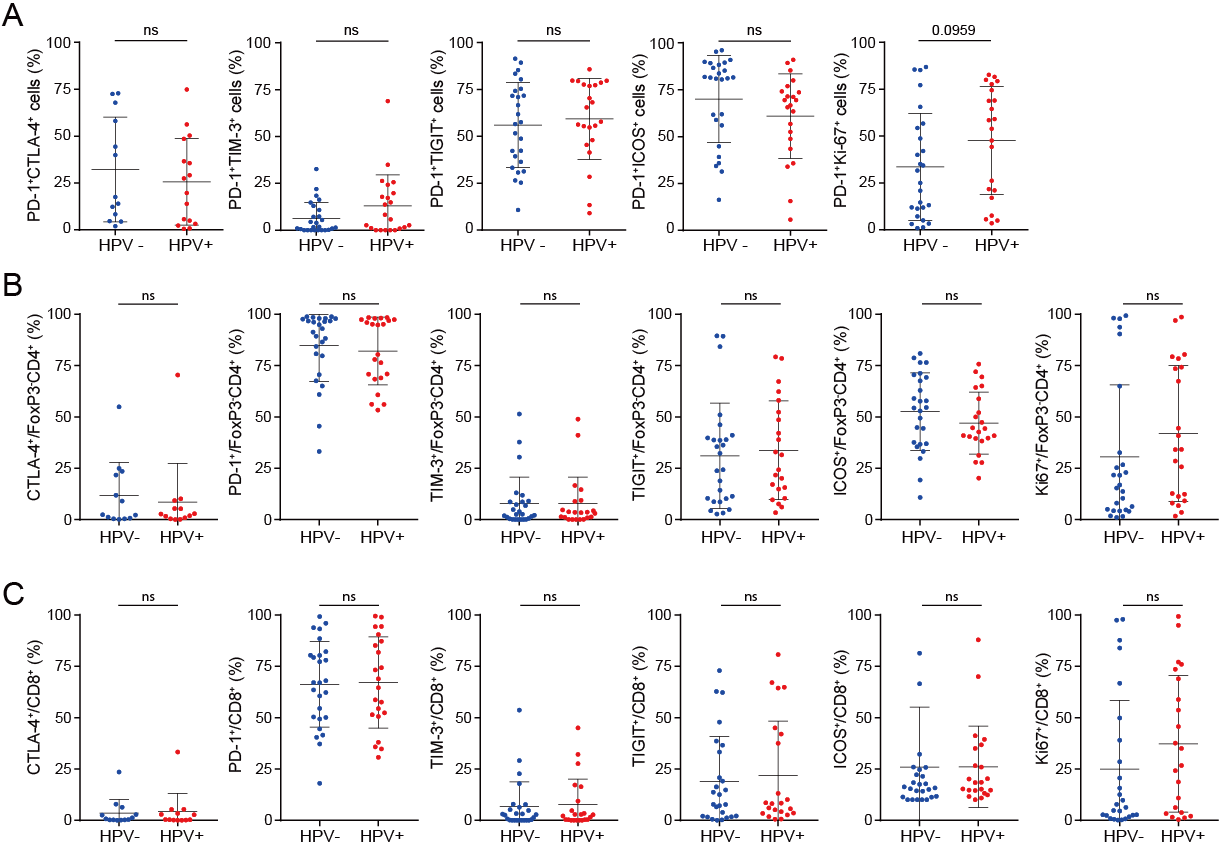
**Figure S7. Phenotypic characteristics of tumor-infiltrating Tregs, FoxP3^-^CD4^+^ T cells and CD8^+^ T cells according to HPV status. (A)** Frequency of PD-1^+^CTLA-4^+^, PD-1^+^TIM-3^+^, PD-1^+^TIGIT^+^, PD-1^+^ICOS^+^ and PD-1^+^Ki-67^+^ cells in the TI FoxP3^+^CD4^+^ Tregs. **(B-C)** Frequency of CTLA-4^+^, PD-1^+^, TIM-3^+^, TIGIT^+^, ICOS^+^ and Ki67^+^ cells in **(B)** FoxP3^-^CD4^+^ T cells and **(C)** CD8^+^ T cells. Phenotypic analysis of each exhaustion**-**and/or activation**-**related markers was analyzed by flow cytometry according to HPV positivity. ns, not significant. All statistical analyses were performed using unpaired Student’s t test.


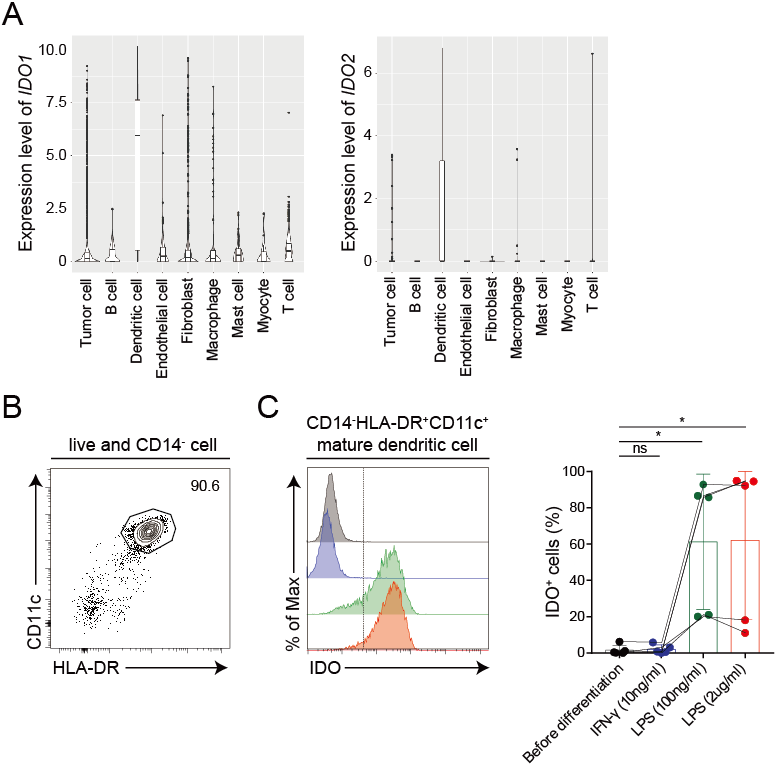


**Figure S8. Expression of IDO-1 and IDO-2 in immune cell subsets. (A)** Public single cell transcriptome analysis of HNSCC (GSE103322) revealed the expression of *IDO1* and *IDO2* in tumor cells and various immune cell subsets. **(B)** Representative plot showing the establishment of mature dendritic cells as indicated by the expression of CD11c and HLA-DR. **(C)** Expression of IDO in the mature dendritic cells upon various stimulation. IDO-expressing cells were upregulated upon LPS stimulation in the mature dendritic cells (left). Frequency of IDO^+^ cells upon IFN-γ (10ng/ml) or LPS (100ng/ml or 2μg/ml) stimulation (right). Grey, before differentiation; blue, stimulation with IFN-γ (10ng/ml); green, stimulation with LPS (100ng/ml); red, stimulation with LPS (2μg/ml). Data shown in (B) and (C) are summarized from five independent experiments. ns, not significant; **P* < 0.05. Statistical analysis was performed (C) using paired Student’s t test.


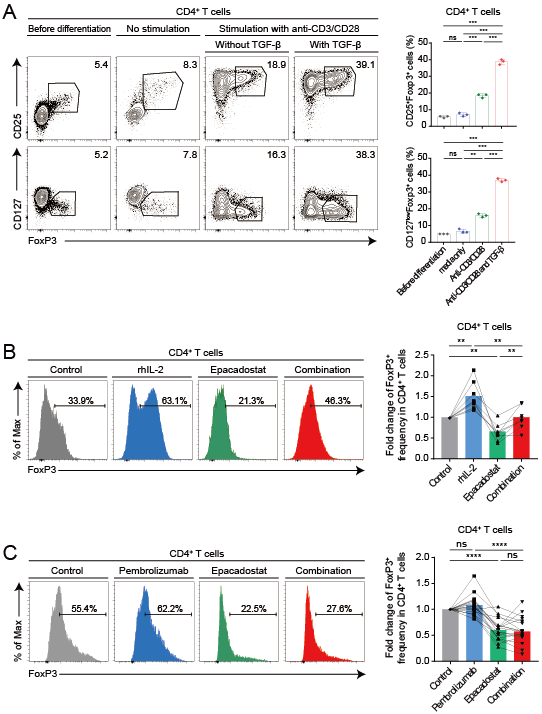


**Figure S9.** Inhibition of IDO with epacadostat and Treg abundancy. (A) Generation of *in vitro*-derived induced Tregs. Representative flow cytometry plots showing the expression of FoxP3 and CD25 (left, upper) and CD127 (left, lower) on CD4^+^ T cells following *in vitro* induced Treg differentiation with or without TCR stimulation and/or TGF-β. Frequency of CD25^+^FoxP3^+^ cells (right, top) and frequency of CD127^low^FoxP3^+^ cells (right, bottom) on CD4^+^ cells after *in vitro* Treg differentiation (N=3). **(B)** Representative histogram displayed the frequency of FoxP3^+^ cells among CD4^+^ T cells further cultured in the presence or absence of rhIL-2 and/or epacadostat followed by the generation of induced Tregs using PBMCs in healthy donor (left). Normalized frequency of FoxP3^+^ cells in CD4^+^ T cells following the various treatments (N=8, right). **(C)** Representative histogram displayed the frequency of FoxP3^+^ cells among CD4^+^ T cells with either the presence or absence of pembrolizumab and/or epacadostat using PBMCs from HPV-positive HNSCC patients (left). Normalized frequency of FoxP3^+^ cells in CD4^+^ T cells following various (N=18, right). Each line in the graph indicates the same sample derived from each individual donor or patient. ns, not significant; ***P* < 0.01; ****P* < 0.001; *****P* < 0.0001. All statistical analyses were performed using paired Student’s t test.


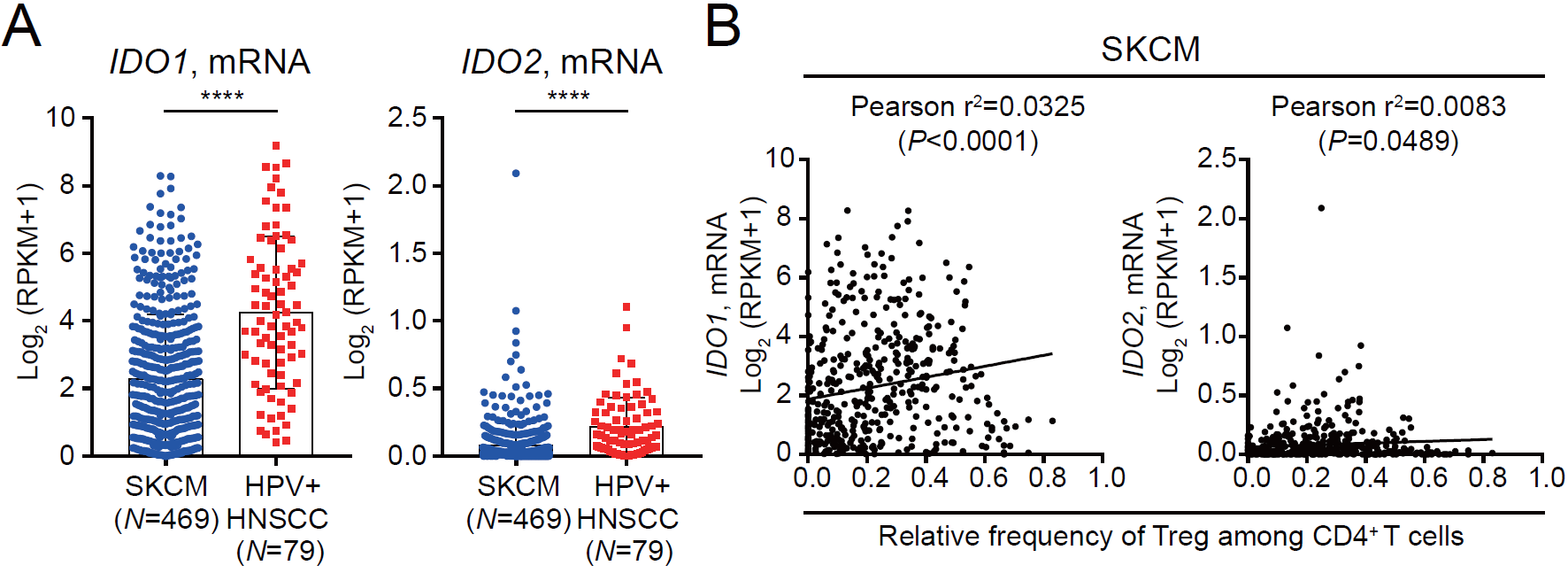


**Figure S10. Comparison of IDO1 and IDO2 expression and their correlation with Treg frequency between TCGA skin cutaneous melanoma (SKCM) and HPV-positive HNSCC cohort. (A)** Comparison of the expression of *IDO1* and *IDO2* from the SKCM cohort and HPV-positive HNSCC cohort. **(B)** Correlation between the relative frequency of Tregs in CD4^+^ T cells and expression of *IDO1* and *IDO2* mRNA in tumor tissues from TCGA SKCM cohort, respectively. *****P* < 0.0001. Statistical analyses were performed (A) using unpaired Student’s t test and (B) using pearson correlation test.


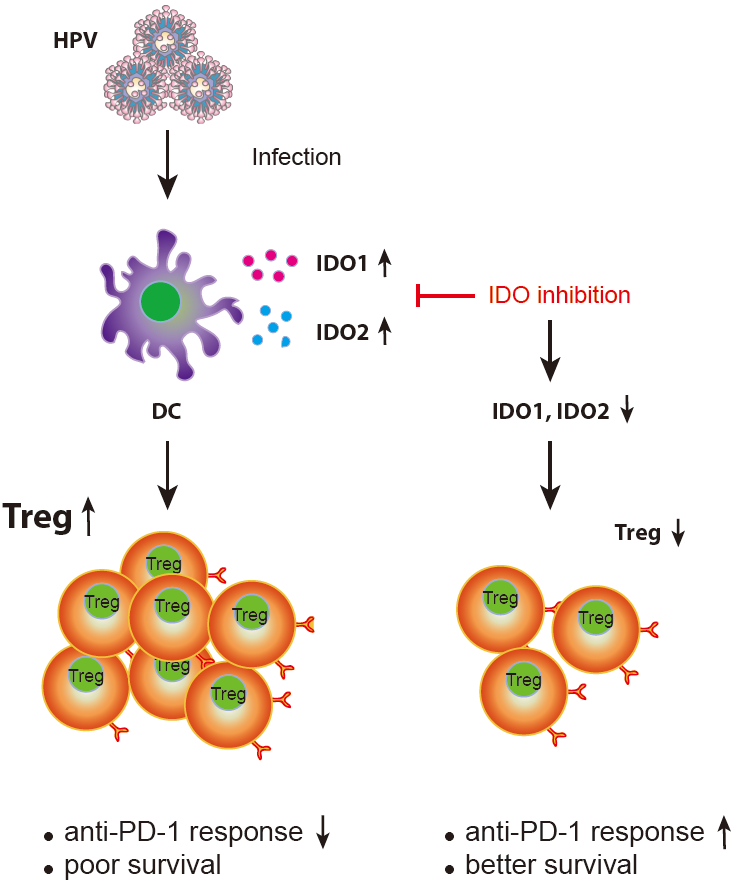


**Figure S11. Graphical abstract**
